# Influence of Two Preparation Techniques on Transportation of Simulated Type II Root Canals 

**DOI:** 10.22037/iej.v13i3.20366

**Published:** 2018

**Authors:** Saeed Moradi, Maryam Gharechahi, Fatemeh Bonyadimanesh

**Affiliations:** a *Dental Materials Research Center, Dental School, Mashhad University of Medical Sciences, Mashhad, Iran; *; b * Dental Research Center, Dental School, Mashhad University of Medical Sciences, Mashhad, Iran;*; c *Private Practice, Mashhad, Iran*

**Keywords:** Apical Transportation, Canal Transportation, Nickel Titanium, Root Canal Preparation, Root Canal Shape

## Abstract

**Introduction:**

The purpose of this study was to evaluate the changes of canal configuration in simulated type II root canals that were instrumented by two different techniques using ProTaper Universal rotary files.

**Methods and Materials:**

Sixty simulated type II root canal in resin blocks were made and randomly divided into two groups. Pre and post-instrumentation images of resin blocks were prepared using stereomicroscope from three surfaces of blocks included two longitudinal section (mesiodistally and buccolingually), and one cross sectional surface. In the first group (G1) the straight canal was instrumented to the working length and the other canal was instrumented up to the area of canals junction. In second group (G2) both canals were instrumented to working length. The superimposed pre and post instrumentation images were assessed by the Adobe Photoshop software. The degree of transportation, centering ability, perimeter, surface and aspetic ratio (AR) in cross section and longitudinal section at apex, 3 mm and 5 mm above the apex, were measured. SPSS software, *t*-test and Mann-Whitney test were used for statistics analysis.

**Result:**

In mesiodistal direction, canal transportation was more (*P*=0.024) only in junction point in G2 which both canals were instrumented to working length. Also, surface changes were more significant (*P*=0.02) in G2 in cross sectional direction. The other parameter and also apical transportation had not significant difference in two groups.

**Conclusion:**

According to the results of this study, it can be concluded that both two preparation methods of type II canals can be used by rotary instruments.

## Introduction

Variety of canal preparation techniques and instruments were designed for adequate canal cleaning and shaping. These stages are regarded the most important aspects of root canal therapy and prerequisite for the success of endodontic treatment [1]. The goals of all these methods include maintaining the original canal morphology, maintaining the foramen size as small as practical, avoiding canal aberrations like ledge formation, zip, and transportation, and creating a conical shape in the direction of crown to apex [2]. Also the clinicians should be noticed that knowledge of the canal morphology is an important requirement for endodontic success. The pulp canal system is complex, and the root canals may branch, divide and rejoin. Weine [3] categorized the root canal system in any root into four basic types. Vertucci [4] found a much more complex canal system and identified eight pulp space configurations. In both grouping methods (Weine and Vertucci), type II is comprised of two separate canals leaving the pulp chamber and joining short of the apex to form one canal [3, 4]. There are two concepts for preparation of type II canals; the first is preparing and obturation of the main canal to the apex and the other canal to the point of juncture. In the second technique, both canals are enlarged to the apex [5].

**Figure 1:**
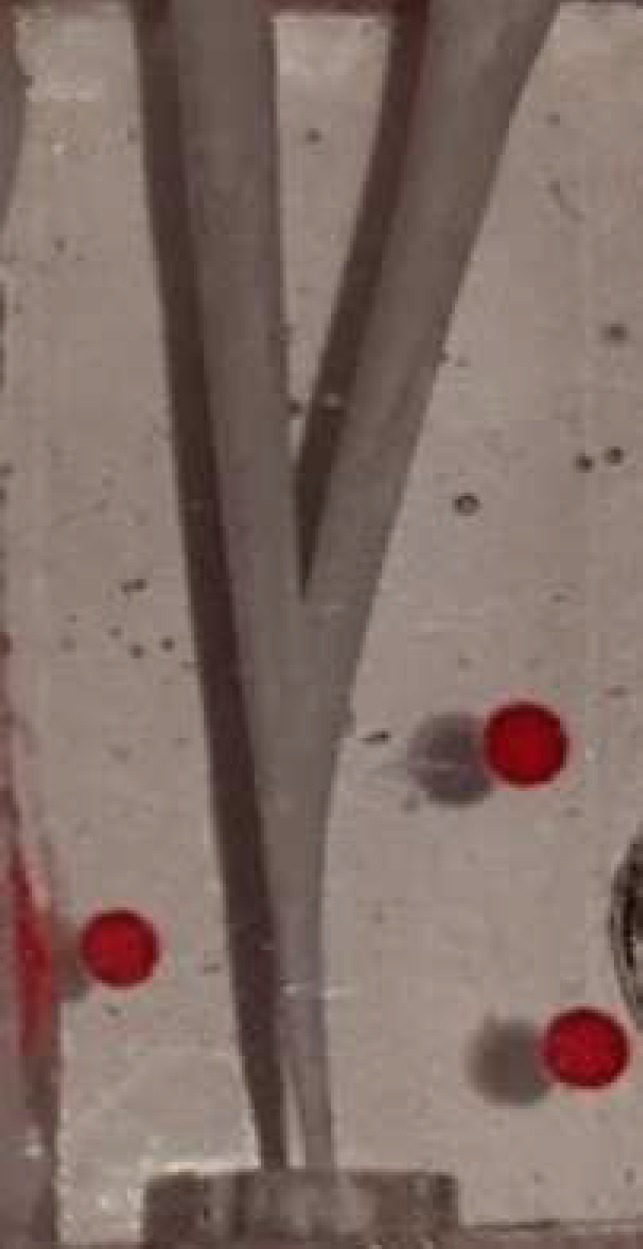
Canal instrumentation in group 2

To decrease these problems and provide the optimum shaped preparation, several instrumentation procedures and instruments have been introduced [6]. Nickel-Titanium (NiTi) rotary instruments have many benefits over hand stainless steel files for example in preserving shape of canal, reducing working time, decreasing operator and patient fatigue and prevalence of technical errors [7].

Various methods, such as double radiographic superimposition technique [8], tooth decalcification [9], CBCT [10-13], and micro CT [14] have been used to investigate preparation errors and root canal transportation. In these studies, the standardization of variables is an important consideration [2]. Radiographic superimposition technique provide only two-dimensional information about root canal morphology [8]. Tooth decalcification allowed effective histological evaluation of the preparation [9], but the destruction of the specimens by the muffle system and decalcification may impede the simultaneous investigation of different parameters of root canal preparation. The recent micro-CT technology allows noninvasive evaluation of both the external and internal morphology of a tooth in a detailed and accurate manner [14].

A serious problem in using teeth in such studies is their inherent variability. Simulated canals in resin blocks have been largely used for assessment of the shaping ability of different NiTi rotary instruments [15]. Weine [3] was the first that used stimulated canals in resin blocks for evaluating the instrumentation effects of endodontic files on root canals.

The purpose of this study is evaluation of changes in shape of simulated type II canals in resin blocks by two different preparation techniques using ProTaper rotary file.

## Materials and Methods

Sixty simulated type II root canal in transparent resin blocks were made from a clear polyester resin (Isophthalic H150-Korea) to assess instrumentation [16, 17]. After mixing with catalyst the casting resin became fluid and could be poured easily into the mold. The blocks were clear after polymerization and their surfaces did not need any polishing. The dental impression material was used for construction of mold for the blocks. Size 20 spreaders with taper of 2% (Mani, Tochigi, Japan) were selected to act as molds for the canals that allowed K-file #15 (Mani, Tochigi, Japan) to negotiate easily the full working length of simulated canals. The two canals were straight without any curvature that jointed to each other with the angle of 20°, and this junction was 5 mm up to apex without any curve. All blocks were 15 mm in length, with 2% taper. Clear casting resin was poured into the mold and allowed to set. Then the spreader was removed 24 h later using plier, leaving a type II canal 15 mm long.

They were randomly divided into two groups of 30 blocks. Three landmarks were provided with a round bur in the three surface of resin block without penetrating into canal. These landmarks ensured an exact matching of pre and post instrumentation images. To calibrate the software that measured the transport, an endometer (Dentsply-Maillefer, Ballaigues, Switzerland) was fixed next to the resin blocks. Pre-instrumentation images of resin blocks in a fixed position were prepared using stereomicroscope (Blue Light-USA), with 10× magnification, from three surfaces of blocks included two longitudinal section (mesiodistally and buccolingually) and one cross sectional surface.

***Preparation of simulated canals***

The instrumentation was performed by a single operator with a handpiece powered using an electric motor control (Endo-Mate DT motor, NSK, Tokyo, Japan). To prepare canals in two groups, ProTaper Universal rotary system (PTU; Dentsply, Maillefer, Ballaigues, Switzerland) was used. ProTaper files (shaping and finishing files) were used in pecking motion as follows; size S1 was advanced to resistance but no more than two third of the canal depth. The SX file was used by brushing action to 3-5 mm short of the working length. After that S1 and S2 were used at the working length. ProTaper finishing files F1and F2 were used at the working length; Files were frequently wiped by using wet gauze to eliminate resin debris. For irrigation of canals, sodium hydrochloride was used through a 31-gauge needle; after use of every instrument also RC Prep (Mynol; Sure Dent Crop, Seoul Korea) was used.

In two groups manual glide path was used with hand instruments during preparation with rotary files. In the first group the straighter canal was selected as the main canal and instrumented to working length, the other canal was instrumented up to the area of canals junction. In second group both canals were instrumented to working length (Figure 1).

**Figure 2:**
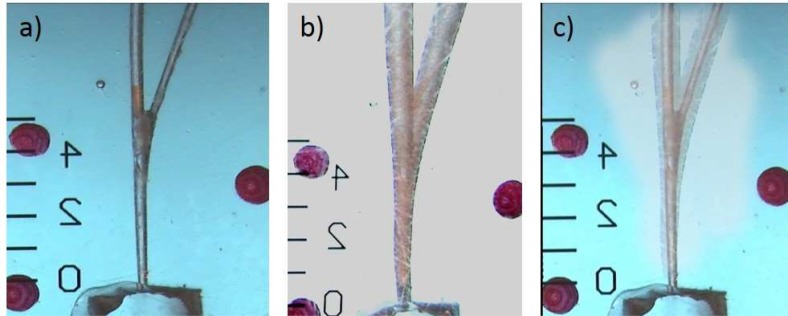
*A)* Pre-instrumentation images of resin block under stereo-microscope; *B)* Post-instrumentation images of resin block under stereo-microscope; *C)* Superimposition of two images

Each resin block was imaged post-instrumentation in the same position as per-instrumentation phase. The landmarks placed in the sides of the resin blocks aided superimposition of the pre and post instrumentation images. The superimposed pre and post instrumentation saved images were assessed by the Adobe Photoshop software (CS6) (Figure 2). The degree of transportation, centering ability, perimeter, surface and aspetic ratio (AR=greater diameter/smaller diameter) in cross section and longitudinal section at apex, 3 mm and 5 mm up to apex were measured.

All of these items were determined by MIP (Microstructural Image Processor) software. The collected data were assessed using SPSS statistical software (SPSS, version 12.0, SPSS, Chicago, IL, USA). All data were checked for normality and presented as mean±standard deviation (SD). Results were analyzed using independent *t*-test students and Mann-Whitney test. *P*-value less than 0.05 was considered as significant.

## Results

In this study, changes in canal surface, perimeter and transportation were evaluated in mesiodistal and buccolingual directions.

***Mesiodistal direction***

In 5 mm point to apex, surface and perimeter changes did not have significant difference in both instrumentation techniques, respectively (*P*=0.136 and 0.167, respectively) but transportation was significantly less in group 1 in comparison with group 2 (*P*=0.024) (Table 1).

In 3 mm distance from the apex, changes in surface and canal transportation were not significantly different in both groups, respectively (*P*=0.766 and 0.812, respectively) but increase in diameter was significantly more in group 2 (*P*=0.010) (Table 1). 

**Table1:** Mean (SD) of surface, displacement, and perimeter of canals in mesiodistal direction in groups 1 and 2

**Increment**	**Group (N)**	**Surface**	**Perimeter**	**Displacement**
**5 mm from the apex (junction)**	G1 (30)	1.125 (0.743)	0.347 (1.840)	0.025 (0.135)
	G2 (30)	1.361 (0.300)	0.452 (0.684)	0.141 (0.237)
***P*** **-value**		0.136	0.167	0.024
**3 mm from the apex**	G1 (30)	0.652 (0.854)	-0.212 (1.159)	0.036 (0.124)
	G2 (30)	0.703 (0.177)	0.452 (0.588)	0.044 (0.128)
***P*** **-value**		0.766	0.010	0.812

**Table 2:** Mean (SD) of surface, displacement, and perimeter of canals in buccolingual direction in groups 1 and 2

**Increment**	**Group (N)**	**Surface**	**Perimeter**	**Displacement**
**5 mm from the apex (junction)**	G1 (30)	0.780 (0.906)	0.125 (2.889)	-0.040 (0.157)
	G2 (30)	1.32 (0.304)	0.168 (0.453)	0.001 (0.173)
***P*** **-value**		0.069	0.941	0.361
**3 mm from the apex**	G1 (30)	0.804 (0.703)	0.740 (0.123)	0.075 (1.872)
	G2 (30)	0.622 (0.132)	0.338 (0.232)	0.0179 (0.147)
***P*** **-value**		0.207	0.303	0.129

**Table 3:** Mean (SD) of displacement of apex in G1 and G2

**Direction **	**Group (N)**	**Displacement**	***P*** **-value**
**Buccolingual **	G1 (30)	0.0662 (0.100)	0.294
	G2 (30)	0.0322 (0.1338)	
**Mesiodistal**	G1 (30)	-0.0085 (0.161)	0.677
	G2 (30)	0.0141 (0.231)	

**Table:** 

**Increment**	**Group**	**Surface**	**Perimeter**	**Centrality**	**AR**
**Cross section**	**G1**	-0.0004 (0.257)	0.183 (0.432)	0.277 (0.093)	-0.054 (0.223)
	**G2**	0.111 (0.205)	0.349 (0.274)	0.266 (0.101)	0.080 (0.292)
***P*** **-value**		0.020	0.093	0.675	0.064

***Buccolingual direction***

In 5 mm and 3 mm point to apex, transportation and changes in surface and perimeter were not significantly different in both instrumentation techniques (Table 2).

***Apex***

Transportation in mesiodistal and buccolingual direction in apex had no significant difference in both groups (*P*=0.677 and 0.294, respectively) (Table 3).

***Cross sectional direction***

Among surface, perimeter, centering ability and AR, the only parameter that had significant difference was surface changes (*P*=0.02) (Table 4).

## Discussion

No laboratory research article is available in relation to the evaluation of the root canal transportation in type II canals in the English literature. So, we conducted a two-part study that evaluated the hand and rotary files in instrumentation of these canals. According to the result of this study, in mesiodistal direction, canal transportation was more in junction point in second group which both canals were instrumented to the working length. Also, surface changes were more significant in group 2 in cross sectional direction.

One of the most common mishaps during the instrumentation of root canals is root canal transportation. As a result, insufficient root canals cleaning and destruction of the root canal integrity occur and residual microorganisms and debris maybe harboring in the apical third of the root canal [6]. Apical transportation more than 0.3 mm can jeopardize the outcome of treatment due to the significant decrease in the sealing ability of root filling material; thus, studies that evaluate apical deviation are important tools to improve clinical practice [18].

The most prevalence of Vertucci’s type II canal (33%) is seen in mesial roots of mandibular molars [3, 4]. There are controversial approach regarding the instrumentation of these canals. Vertucci believes that this anatomy is best treated by preparing and obturating the main canal to the apex and the other canal to the point of juncture. If both canals are enlarged to the apex, an hourglass preparation may results; the point where the two canals join is more constricted than the preparation at the apex [4]. Filling such a configuration leaves voids in the apical third, favoring treatment failure, particularly if microorganisms or their byproducts remaining in the canal [5]. According to this study, there was no significant difference between the two preparation techniques in causing apical transportation in buccolingual and mesiodistal directions in type II canals. Assessment of canal changes in both buccolingual and mesiodistal directions was performed with the purpose of describing the three-dimensional morphological changes during root canal preparation. The point where the two canals join is more transported in mesiodistal directions in group that both canals were instrumented to working length. Also the less transportation was seen in the apex point in compression to the other increments. So, in contrast to the Vertucci’s concept, the hourglass configuration was not caused in this study. It seems that the canal displacement at the junction is not as important as the apical transportation. Another finding of this study was that the amount of apical transportation less than 0.3 mm that can jeopardize the endodontic outcome according to the study by Nazari *et al.* [18]. Other study should be designed to obturated these canals and evaluated the effect of these results on the seal of the canal. The result of part 1 of this study was as follows; when these canal configurations were instrumented with hand files, both preparation techniques caused transportation in both mesiodistal and buccolingual directions and there were no significant differences between the two techniques in causing transportation in both buccolingual and mesiodistal directions in each increments [5].

The use of simulated canals is particularly useful when investigating instrumentation of the canals because it is nearly impossible to select human teeth with type II canals that have similar parameters in terms of canal length, diameter, and degree and radius of both branches. So, we can design the study with proper sample size. These simulated canals can be easily photographed, measured and evaluated before and after canal preparation [7, 15]. However, the results of studies using simulated canals must be extrapolated cautiously to clinical conditions because of the differences that exist between resin and dentin [15].

## Conclusion

Under the condition of this study, it can be concluded that rotary instruments can be used in type II canals in both preparation methods.
